# Uptake and predictors of colonoscopy use in family members not participating in cascade genetic testing for Lynch syndrome

**DOI:** 10.1038/s41598-020-72938-z

**Published:** 2020-09-29

**Authors:** Donald W. Hadley, Dina Eliezer, Yonit Addissie, Andrea Goergen, Sato Ashida, Laura Koehly

**Affiliations:** 1 Office of the Clinical Director, National Human Genome Research Institute, National Institutes of Health, 35 Convent Drive, MSC 3717, Bldg. 35, Room 1B205, Bethesda, MD 20892-3717 USA; 2 Social Networks Methods Section, Social and Behavioral Research Branch, National Human Genome Research Institute, National Institutes of Health, Bethesda, MD USA; 3 Department of Community and Behavioral Health, College of Public Health, University of Iowa, Iowa City, IA USA

**Keywords:** Cancer genetics, Genetic testing, Colonoscopy, Preventive medicine, Colon cancer

## Abstract

Cascade genetic testing provides a method to appropriately focus colonoscopy use in families with Lynch syndrome (LS). However, research suggests that up to two-thirds at risk to inherit LS don’t participate. Within the United States, no studies have assessed colonoscopy use within this elusive and high-risk subset. We set forth to (1) document colonoscopy use within those not undergoing genetic testing (NGT) and (2) identify factors associated with completing colonoscopy. Data came from a cross sectional survey of families with molecularly confirmed LS. One hundred seventy-six (176) adults participated; 47 of unknown variant status and 129 with variant status known (59 carriers/70 non-carriers). Despite a high level of awareness of LS (85%) and identical recommendations for colonoscopy, NGT reported significantly lower use of colonoscopy than carriers (47% vs. 73%; *p* = 0.003). Our results show that perceived risk to develop colon cancer (AOR = 1.99, *p* < 0.05) and physician recommendations (AOR = 7.64, *p* < 0.01) are significant predictors of colonoscopy use across all family members controlling for carrier status. Given these findings, health care providers, should assess patients’ perceived risk to develop cancer, assist them in adjusting risk perceptions and discuss recommendations for colonoscopy with all members in families with LS.

*Trial Registration* Clinical Trials.gov Identifier: NCT00004210.

## Introduction

Lynch syndrome (LS) is an inherited cancer susceptibility syndrome caused by inactivating variants in one of four mismatch repair genes (MSH2, MLH1, MSH6, PMS2)^1^ or in the EPCAM gene^2^. LS is the most common inherited cause of colorectal cancer (CRC) accounting for 2–4% of CRC cases. Lifetime risks for developing CRC approach 75% in men and 50% in women^3^. Women also have lifetime risks for endometrial cancer equivalent to their risks for CRC. In addition, individuals carrying pathogenic variants in LS associated genes are at increased risk for a broad spectrum of other cancers including stomach and ovarian cancers^3^.

It is estimated that between 1 in 100^4^ and 1 in 279^5^ persons carry a pathogenic variant for LS. Based upon the U.S. Census data for 2010, this translates into a range of people at increased risk for the early onset of cancers associated with LS between 1 and 3 million. The identification of these individuals prior to the occurrence of disease provides for targeted screening preserving resources for those at high risk of developing cancer, avoids unnecessary procedural risks while providing reassurance for those testing negative^6^. Previous research demonstrates clear medical benefits from early detection and screening for CRC associated with LS reporting a 62% reduction in the incidence of CRC^6^ and a 65–72% decrease in mortality in families with a LS-associated variant^7–9^. From a behavioral perspective, research reveals significant and appropriate changes in colonoscopy use following genetic counseling and testing; carriers increase colonoscopy use and non-carriers significantly decrease use^10,11^. While research has not shown a psychological benefit, it has shown that adaptation to genetic results seemingly occurs without lasting, harmful psychological sequela in those utilizing genetic services^12–14^.

If a pathogenic variant is identified in families suspected to have LS, cascade genetic testing is recommended within the family following the provision of genetic counseling. As noted, cascade genetic testing allows for the differentiation of family members at high risk of developing LS cancers (carriers) that require earlier and periodic screening from those at population risk (non-carriers). However, the medical and economic benefits of cascade genetic testing depend on maximizing uptake of genetic testing by at risk relatives while ensuring adherence to recommended cancer screening guidelines^15,16^. Both parameters present challenges and would benefit from additional research to maximize uptake and improve adherence to recommendations for cancer screening in families with LS.

### Uptake of genetic testing in families with pathogenic variants associated with LS

A systematic review of the literature on LS^17^ reports that up to 66% of first-degree relatives (parent, child or sibling) do not undergo genetic testing following the identification of a pathogenic variant in the family. Furthermore, the literature suggests that the uptake of genetic testing in more distant relatives (second and third-degree relatives) is significantly lower than in close relatives^18^. Collectively, these studies indicate that a clear majority of family members are not utilizing genetic services to clarify their risk for developing a LS cancer. Not surprisingly, LS does not stand alone in this statistic as a number of studies have reported lower than expected uptake of genetic testing within families facing other dominantly inherited diseases with options available to prevent the disease or provide early detection^6,19,20^.

A number of factors have been reported and associated with the uptake of genetic testing in LS^18,21,22^. The list includes demographics factors, psychological wellbeing (lack of depressive symptoms), family history (number of relatives with cancer), parental adherence to regular screening, being an only child, being a sibling of a tested carrier, family communication and social networks interactions within families.

### Colonoscopy use within families with pathogenic variants

Since the published literature has consistently focused on family members who receive genetic services and undergo testing, the screening behaviors of the silent majority, i.e. family members not utilizing genetic services, have been ignored. Of concern is the absence of data on their use of colonoscopy given the proven benefits of interval screening in families with LS.

Persons who are at risk to inherit a pathogenic family variant but do not undergo genetic testing to clarify their cancer risk status are recommended to screen for CRC at the same ‘high’ level as carriers, i.e. colonoscopy every 1–2 years beginning no later than 25 years of age or 2–5 years before the youngest diagnosis of colorectal cancer if occurring before 25 years of age^23^.

However, investigations into the cancer screening behaviors of family members at risk to inherit a LS associated variant but not utilizing genetic services are scarce. A study from the United Kingdom (UK) utilized a regional LS registry to explore colonoscopy use^24^. That study reported greater than 97% colonoscopy use within the 2 years prior to the study among first-degree relatives identified as variant carriers and 35% completion of colonoscopy by family members not utilizing genetic services. The reported use by variant carriers in the UK is notably higher than reports from US studies and may be the result of their system of nationalized health care.

The only US study to report on colonoscopy use in family members opting to forego variant testing occurred after the receipt of comprehensive information about Lynch syndrome which included recommendations for colon cancer screening^22^. Within that study, variant carriers underwent colonoscopy within the 12 months that followed the receipt of genetic results significantly more often (73%; 16/22) than family members at risk to inherit their family variant, but opting out of testing (22%; 6/27). While this study is quite small, additional research seems prudent given an ever-increasing recognition that a significant majority of family members don’t receive genetic education, counseling and don’t participate in cascade genetic testing.

Certainly, part of the equation to improve colonoscopy use relies on the identification of factors that influence that behavior. Variant status has been consistently recognized as a variable influencing colonoscopy use for those undergoing testing^10^. Likewise, patient-provider communication regarding genetic test results and encouragement to screen has been shown to influence cancer screening following the identification of a pathogenic variant associated with LS^24–28^. However, the literature is lacking evidence of the impact of patient-provider communication in the presence of a family history of molecularly confirmed LS but in the absence of a genetic test results.

Perceptions of one’s risk to develop colon cancer have also been identified as a variable influencing colonoscopy use in families with colon cancer as well as families with LS specifically^25,26,29,30^ with noted exceptions^31^. Family members who receive genetic services and are informed of their variant status alter their initial risk perceptions appropriately with carriers reporting higher perceived risk than non-carriers following the receipt of genetic services^25,32^. Further, those who undergo variant testing complete colonoscopy in keeping with their variant status over the short term; carriers increase use and non-carriers significantly decrease use of colonoscopy. However, research suggests that individuals generally have difficulty comprehending the concept of disease risk^25,33,34^ and specifically have difficulty quantifying risk accurately^35^. Without the clarification of risks (genetic and cancer risks), review of cancer risk reducing strategies, and discussion of experiences and perceptions regarding cancer that typically occur through the provision of genetic counseling, it is feasible that family members not utilizing genetic services may perceive their risk for developing colon cancer at levels too low to motivate preventive health behaviors^17^.

Unfortunately, the existing literature is devoid of insights regarding the large and relatively ignored subset of family members at risk to inherit an identified pathogenic variant but not receiving genetic counseling nor undergoing genetic testing. Research efforts are needed to assess cancer screening behaviors within this population as well as to identify modifiable variables that can be used to influence behavior within our existing health care system. Therefore, this study was designed to (1) assess colonoscopy use in family members not utilizing genetic services; (2) assess the role of perceived risk in colonoscopy use and (3) explore the role of provider recommendations in influencing colonoscopy use.

## Results

### Participant characteristics

The majority of the study sample was white (97%), female (61%), health insured (92%), college educated (55%), married or partnered (65%), employed (72%), and lacking a history of cancer (75%). Ages ranged from 18 to 87 years with an average age of 46 years. GT+ were more likely to have a personal history of cancer than GT− (*p* < 0.001).

The subset of family members who were at risk to inherit the family variant but did not receive genetic counseling nor undergo genetic testing (NGT; n = 47) was composed of:

- Seventeen family members (17/47; 36%) whose parent was identified as a carrier (Mendelian risk = 50%);
- Twenty-six family members (26/47; 55%) whose parent, at-risk to inherit the familial variant, was deceased at the time of the study (Mendelian risk = 25%). Twenty-one of the 26 deceased parents were reported to have had a LS associated cancer at less than 55 years of age and
- Four family members (4/47; 8.5%), whose living, at-risk parent also chose not to utilize variant testing (Mendelian risk = 25%). Three of five (3/5; 60%) of these parents were reported to have a personal history of cancer.

Eighty-five percent (40/47) of the NGT group reported that they were aware that family members were taking part in a study about LS; 74% (35/47) spoke directly with family members about being involved in the LS study. Seven (7/47; 15%) reported that they were neither aware of the study nor spoke to a family member about the study prior to being invited to complete the current survey.

Table 1 provides a comparison of socio-demographic and other variables of interest stratified by variant status. Reported *p* values were made for comparisons between GT+ and NGT since recommendations for colonoscopy use are identical for these family members.

**Table 1:** Demographic characteristics, physician recommended colonoscopy, perceived risk, wellbeing, and colonoscopy screening by variant status (n = 176).

Characteristic	Variant carriers GT+	Non-carriers GT−	Unknown variant status NGT	*p* value* (GT+ vs. NGT)
	(n = 59)	(n = 70)	(n = 47)	
Age	Mean = 44 years	Mean = 47 years	Mean = 45 years	*p* = 0.88
	S.D. = 13 years	S.D. = 15 years	S.D. = 17 years	
	Range = 21–71 years	Range = 23–81 years	Range = 18–83 years	
% Female	64%	61%	57%	*p* = 0.30
% having/had cancer	37%	14%	28%	*p* = 0.35
% health insurance	93%	94%	87%	*p* = 0.32
% physician recommended colonoscopy	90%	73%	79%	*p* = 0.06
% College educated	54%	63%	45%	*p* = 0.60
% Employed	78%	69%	70%	*p* = 0.36
Perceived risk of colon cancer	Mean = 4.66	Mean = 3.24	Mean = 3.77	*p* = 0.001
	S.D. = 0.73	S.D. = 0.79	S.D. = 1.40	
	Range = 1–5	Range = 1–5	Range = 1–5	
CESD score	Mean = 3.78	Mean = 4.56	Mean = 6.88	*p* < 0.001
	S.D. = 4.03	S.D. = 5.13	S.D. = 5.10	
	Range = 0–15	Range = 0–26	Range = 0–24	
Colonoscopy use	73%	36%	47%	*p* = 0.003

### Colonoscopy use

**Figure 1:**
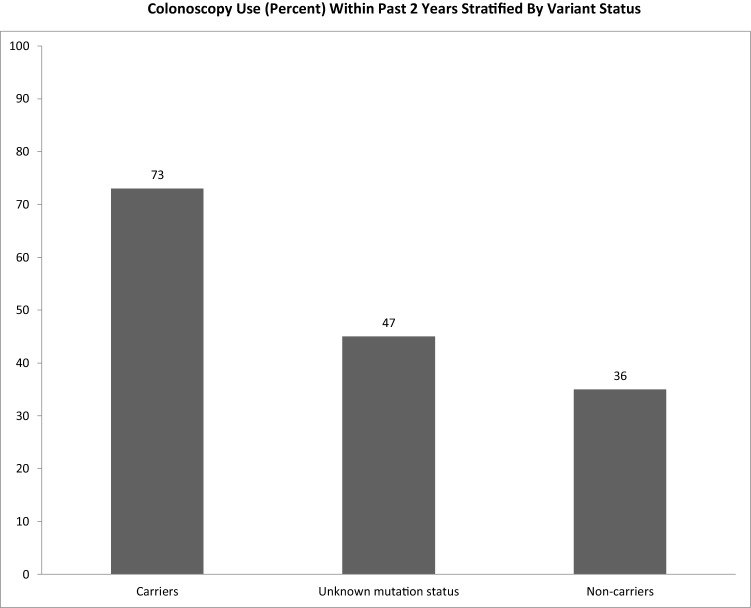
Colonoscopy use (percent) within past 2 years stratified by variant status.

Overall, 73% of GT+ underwent colonoscopy within the 2 years prior to completing the survey, compared to 47% of NGT and 36% of GT− (Fig. 1). Controlling for demographic characteristics, personal cancer history, and psychological wellbeing, GT+ were nearly 4 times more likely (Adjusted Odds Ratio [AOR] = 4.02, *p* = 0.006) to have had a colonoscopy than not during the specified time compared to NGT. However, NGT did not significantly differ from GT− in their use of colonoscopy (AOR = 1.83, *p* = 0.15). Consistent with the reported literature, GT+ were 6 times more likely than GT− to have had a colonoscopy than not within the within the 2 years prior to the assessment (AOR = 6.36, *p* < 0.001). Age was the only significant covariate; age was positively associated with colonoscopy use (AOR = 1.04, *p* = 0.008).

### Provider recommendation for colonoscopy

There is a marginal association in provider recommendation for colonoscopy by testing status, with 90% of GT+, 70% of GT−, and 73% of NGT reporting receiving such recommendations (*p* = 0.06). Controlling for covariates, GT+ are significantly more likely to report provider recommendations for colonoscopy than NGT (AOR = 6.56, *p* = 0.03), with no difference in referrals between NGT and GT− (AOR = 1.30, *p* = 0.70). Age was the only significant covariate (AOR = 1.08, *p* < 0.001).

### Perceived risks to develop colon cancer

NGT had significantly lower perceived risk of developing colon cancer (*p* = 0.001) than GT+. Results remain consistent when controlling for covariates. Those with health insurance reported significantly lower risk perceptions (*p* = 0.027) as well.

### Multivariate analysis

As stated, NGT perceived themselves to be at lower risk of developing colon cancer compared to GT+. Mediation analysis was conducted to explore whether these lower risk perceptions explain NGT’s lower rates of colonoscopy screening compared to GT+. Provider recommendation partially mediates the association between testing status and colon cancer screening (Model 2a: AOR = 10.65, *p* < 0.001) and is a strong, consistent predictor of colonoscopy use when perceived risk is added to the model (Model 2b: AOR = 7.64, *p* < 0.001). Perceived risk for developing colon cancer was a significant predictor (AOR = 2.05, *p* = 0.004) of colonoscopy use suggesting that risk perception may play a role in how knowledge (or lack of knowledge) regarding one’s variant status may impact screening behavior. Inclusion of both physician referral and perceived risk appears to fully explain observed differences in colonoscopy use between NGT and GT+ (Table 2).

**Table 2:** Multivariate logistic regression, predicting colonoscopy use within 2-years of assessment (n = 176).

	Colonoscopy use (Model 1)	Colonoscopy use (Model 2a)	Colonoscopy use (Model 2b)
	OR (95% CI)	OR (95% CI)	OR (95% CI)
**Covariates**			
Age	1.05** (1.02; 1.08)	1.04* (1.00; 1.07)	1.05* (1.00; 1.09)
Male	1.08 (0.51; 2.27)	1.22 (0.54; 2.76)	1.41 (0.66; 3.04)
Has health insurance	1.01 (0.31; 3.33)	0.92 (0.15; 5.56)	1.09 (0.17; 6.99)
College educated	1.58 (0.79; 3.15)	1.58 (0.76; 3.27)	1.52 (0.72; 3.22)
Employed	1.00 (0.43; 2.32)	1.06 (0.44; 2.56)	0.96 (0.35; 2.61)
Depressive symptoms	1.03 (0.98; 1.08)	1.01 (0.95; 1.08)	1.00 (0.94; 1.07)
Cancer history	1.49 (0.55; 4.02)	1.38 (0.52; 3.64)	1.32 (0.48; 3.60)
**Genetic testing**			
GT+ (carrier)	Referent	Referent	Referent
GT− (non-carrier)	0.15*** (0.08; 0.30)	0.18*** (0.09; 0.34)	0.40* (0.17; 0.96)
NGT (status unknown)	0.24** (0.08; 0.69)	0.34* (0.12; 0.99)	0.53 (0.20; 1.37)
Physician colonoscopy referral		10.65*** (3.08; 36.76)	7.64** (1.85; 31.59)
Perceived risk			1.99* (1.14; 3.47)

Generalized estimating equations, with exchangeable covariance.

*p <  0.05; **p <  0.01; ***p < .0.001.

## Discussion

The existing literature indicates that less than half (23–45%) of first degree relatives at risk for inheriting Mendelian (monogenic) diseases with risk reducing or preventive strategies, utilize genetic services to guide their medical management; these data include families with Lynch syndrome^17,19^. However, published research has focused on family members who receive intensive genetic education, counseling and undergo testing, and as such, neglect the behavioral and psychological outcomes of the ‘silent majority’ of family members who don’t seek genetic services. To our knowledge, the present report is the first US study to document colonoscopy use by family members at risk for inheriting Lynch syndrome but not utilizing genetic services.

Our data show that less than half (47%) of family members not participating in genetic testing complete colonoscopy within the 2 years prior to their participation in this study compared to 73% of relatives known to carry the pathogenic familial variant. This is a significant difference in colonoscopy use and occurs despite identical recommendations for colon cancer screening. Ideally, improving the uptake of genetic testing within families would allow for more targeted and efficient use of cancer screening resources. However, in the current climate of low uptake of genetic testing and significantly reduced levels of colonoscopy by at risk family members, professional societies are promoting a ‘better safe than sorry approach’ to colon cancer screening while acknowledging the inefficiencies and associated medical risks of undergoing a potentially unnecessary colonoscopy. Ultimately, additional efforts are needed to improve the uptake of cascade genetic testing in LS and a host of other inherited diseases/disorders with proven strategies to improve health outcomes.

A recent systematic review of the literature on the uptake of pre-symptomatic genetic testing in hereditary breast-ovarian cancer and Lynch syndrome found that genetics centers that directly contacted at risk relatives reported a higher number of relatives undergoing testing compared to a proband mediated approach, i.e. relying on participating family members to share the information with at risk relatives^36^. However, direct contact of family members by health care professionals poses additional concerns from social and legal perspectives that tend to hinder the use of this approach on a broad scale^37,38^.

As we report, provider recommendation regarding colonoscopy is a strong, consistent predictor of colonoscopy use when perceived risk was included within the model, highlighting the importance of open communication among patients and providers related to their family history of LS. Therefore, efforts to improve colonoscopy use within families with LS will best be addressed through enlisting both genetics providers who encounter individuals pursuing genetics services and health care providers encountering family members more broadly (primary health care providers as well as specialists). In both situations, health care providers have the opportunity to assess perceptions of disease risk regardless of variant status. In the case of persons who have undergone testing and know their carrier status, reviewing cancer risks associated with LS and cancer screening guidelines are certainly appropriate. However, and in addition, conversations about personal perceptions of developing cancer may provide opportunities to assess and clarify misperceptions potentially arising from subjective experiences occurring within the family. In situations where the patient’s subjective risk does not match the objective risk (either molecularly determined or Mendelian risk), both education about cancer risk and encouragement by health care providers has been shown to improve cancer screening^25,38^.

In situations where health care providers encounter family members at risk for inheriting LS but have not engaged in genetic services, the collection and review of the family medical history provide opportunities to discuss both Mendelian risks to inherit LS and the associated cancer risks. As part of that process, health care providers can assess the patient’s perception of their risks to develop colon cancer as well as other potential barriers. Patient responses may provide insights useful in aligning subjective risks with objective risks with the ultimate goal of improving compliance with recommendations for colonoscopy even in the absence of genetic testing. Previous research has shown that preventive messages tailored to persons with a family history of colorectal cancer have been shown to be effective at increasing disease risk perceptions among those who underestimate their risk for colon cancer^39^. It is plausible that a proportion of patients come to the clinical encounter with limited understanding of their family history of LS and the associated risks. Likewise, it is also possible that family members who don’t utilize genetic services aren’t sharing the family history and/or genetic test results of family members with their provider. As such, providers may not be aware of their patients’ potentially increased risk for developing cancer. Furthermore, in the absence of a genetic test result, providers may not know what the recommendations are for colonoscopy. All of these scenarios point to additional need to promote effective family communication about genetic risks and facilitate open communication among families, patients and providers.

In regards to the potential of utilizing health care providers to motivate screening, there appears to be an underlying assumption that health care providers understand the hereditary and lifetime cancer risks associated with Lynch syndrome, know the cancer screening guidelines recommended for individuals within families with LS and accept a role in identifying and managing patients in families with LS. Unfortunately, existing literature within and outside of the United States raises doubt about those assumptions. A study conducted in Australia assessing clinicians’ knowledge, attitudes and referral patterns of patients with suspected Lynch syndrome^40^ reported that 30% of physicians did not feel that their role is to identify patients for genetic referral. A second study by a group from Denmark^41^ investigated knowledge about key features of LS in at risk individuals and physicians in surgery, gynecology and oncology. Within this study, only half of the family members and one third of the physicians correctly estimated the risk to inherit a LS predisposing mutation. Furthermore, physicians generally underestimated the risk of LS associated cancers and three out of four suggested a later starting age for surveillance than recommended. Additional evidence is provided from a US study^42^ that focused on determining comfort and knowledge among obstetrician/gynecologists and general surgeons regarding recommendations for cancer screening for women with Lynch syndrome. That study reported that obstetrician/gynecologists were more comfortable than general surgeons with counseling patients on endometrial cancer screening but less comfortable counseling patients on colon cancer screening. Of interest, there was no correlation between a physician's perceived knowledge and number of correct answers. While more current studies would be informative, these studies illustrate the challenge health care providers face in caring for patients in families with LS.

There is an additional and worrisome finding that we would like to draw attention to reported in Table 1. Within the subset of family members not undergoing genetic testing (NGT), we identified significantly higher numbers of self-reported depressive symptoms compared to family members identified as variant carriers (*p* < 0.001). While depressive symptoms were not found to be associated with screening behavior, our report is the second study to identify increased numbers of depressive symptoms within the subset of family members not undergoing genetic testing within the framework of cascade genetics testing for Lynch syndrome^22^. While the actual scores did not consistently reach a level of clinical significance, this finding is worrisome and could potentially contribute to the lower uptake of genetic services (education, counseling and testing) within families with LS and potentially families confronting other inherited diseases/disorders. While further research is needed, this data provides additional support for implementation of the 2016 recommendations put forth by the United States Preventive Services Task Force (USPSTF) on screening adults for depression especially in families with inherited diseases/disorders.

While we are enthusiastic about the importance of our findings, we acknowledge that there are limitations within the current study. First and of significant concern, is the fact that our cohort does not adequately represent the racial, ethnic or socioeconomic diversity of families with LS or the general population within the United States. Future studies addressing this issue should focus on recruiting a more diverse and representative sample to broaden the usefulness of results. Second, while our sample size of family members at risk to inherit a LS variant but not utilizing genetic services (NGT) is admittedly small, the population is difficult to recruit and engage as evidenced by the lack of published research on this topic. In addition, our method of recruiting NGT through participating family members is likely to have biased the sample completing surveys, possibly recruiting family members more engaged in family communication, less resistant to genetic risk information, generally less distressed and possibly more engaged in cancer screening. Given this likely recruitment bias, outcomes from a larger, more inclusive sample would possibly result in more worrisome outcomes.

In closing, we hope this research serves to engage not only genetic health care providers in the care of this seemingly ignored subset of family members at risk for LS but also recruits health care providers encountering them in other health care settings. Reservations about and initial decisions to not pursue genetic services may change as situations within the family evolves; e.g. new cancer diagnoses within the family, successful outcomes due to the early diagnosis of cancer, change in employment/insurance status, parenthood, etc. At these times, revisiting family experiences, personal feelings, risk perceptions, knowledge of LS, options for testing, and cancer screening, may increase the uptake of cascade genetic testing and focus cancer screening on those truly at increased risk to develop cancer.

## Patients and methods

### Sample

**Figure 2:**
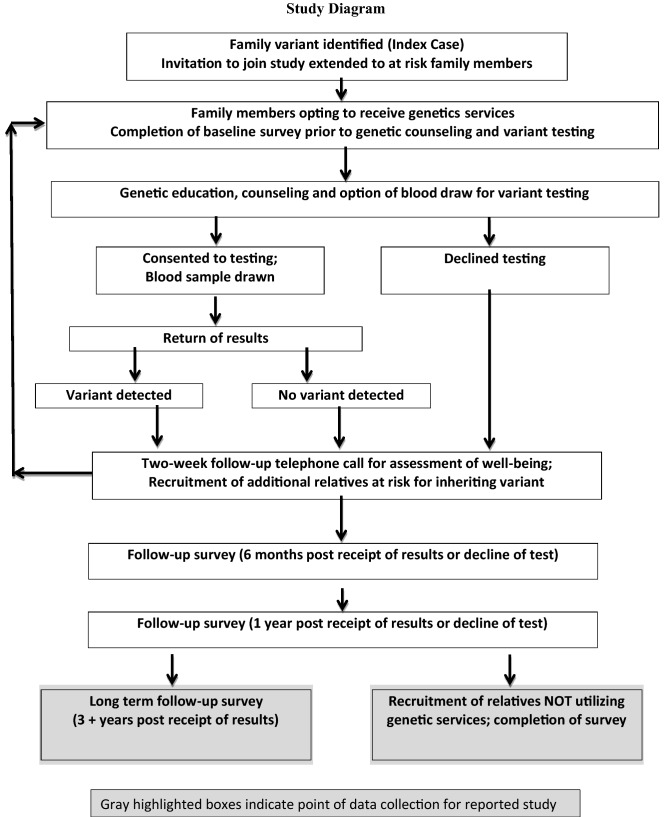
Study diagram.

Study participants were drawn from a cohort of 52 families from 34 of the continental United States with clinically confirmed, pathogenic variants associated with LS. Family members at 50% risk to inherit a pathogenic variant were offered participation in a prospective study investigating the psychological, social and behavioral outcomes of genetic counseling and the option of variant testing^43^. In situations where the ‘at risk’ connecting relative was deceased or declined genetic services, invitations were extended to second degree relatives of known variant carriers (25% Mendelian risk). Within this cohort, 259 family members at risk to inherit their family’s variant participated in genetic counseling, underwent genetic testing, received test results (carrier [GT+] or non-carrier [GT−]) and completed the initial study surveys between 1997 and 2008. Nearly half (49%) of first-degree relatives at risk to inherit the family variant did not participate^44^ suggesting even higher numbers of more distant relatives also at risk for LS but not utilizing genetic services^44^. The sample considered within the current report includes (1) family members receiving genetic counseling and undergoing genetic testing [GT] who completed all questions pertaining to the variables of interest within the long-term follow-up assessment (59 GT+ and 70 GT−) as well as (2) family members at risk to inherit the family variant but not receiving genetic counseling nor undergoing genetic testing [NGT] (n = 47). Recruitment and data collection of NGT occurred simultaneous with the long-term prospective follow-up of cohort participants who underwent genetic testing (Fig. 2).

### Procedures

The Institutional Review Board at the National Human Genome Research Institute of the National Institutes of Health in Bethesda, Maryland approved these investigations; NIH protocol 95-HG-0165, Outcomes of Education and Counseling for HNPCC/Lynch Syndrome Testing (https://clinicaltrials.gov/ct2/show/NCT00004210?term=lynch+syndrome+and+bethesda&rank=2; 01/27/2003). All research was performed in accordance with all guidelines and regulations required by the National Human Genome Research Institute at the National Institutes of Health. Detailed descriptions of the recruitment, genetic education, counseling and offer of variant testing have been previously reported^45^. In brief, family members opting to receive genetic services provided written consent for their longitudinal participation. Participants who elected to receive genetic counseling and consider genetic testing completed surveys at baseline (prior to receiving genetic education and counseling), at 6 months and 1 year after receiving their variant results. A final ‘long term’ follow-up survey was mailed to family members undergoing genetic testing (GT) yielding information from 137 participants 3 to 8 years after their receipt of variant results (Fig. 2).

One hundred forty-eight (148) potential NGT participants were identified and provided a paper questionnaire and postage paid return envelope through GT family members who completed a long-term follow-up survey. For NGT choosing to take part in the study, data were collected simultaneous to that of GT who completed the final long-term follow-up survey. Consent for NGT was implied by the completion and return of the questionnaire. A $50 gift card was provided for the completion and return of the GT long-term follow up and NGT survey. Sixty-four (64) surveys were completed and returned (43% response rate) by NGT at risk for inheriting the identified family variant. Thirteen (13) of these surveys were excluded due to the participants’ reported receipt of genetic services elsewhere and knowledge of their variant status; this left fifty-one (51) NGT whose variant status was not known for the final analyses. Responses to the GT long-term follow up and the NGT survey are the focus to the current report; 12 participants were missing data on key variables, resulting in a total sample size n  =  176 (59 GT+, 70 GT−, 47 NGT).

### Variables

The primary outcome in the current report is colonoscopy use in the 2 years prior to assessment. Participants’ self-reported use of colonoscopy was elicited from their response to a single question asking when they had last undergone colonoscopy. Responses included: (1) within the last year; (2) within the last 2 years; (3) more than 2 years ago, or (4) never. Responses to colonoscopy use were dichotomized into (1) within the last 2 years (responses 1 and 2) or (2) more than 2 years/never (responses 3 and 4).

The primary predictor variable of interest is whether the participant underwent genetic testing (GT or NGT). For those receiving genetic services, categorization of variant status (GT+ or GT−) was based on test results conducted within a laboratory approved by the Clinical Laboratory Improvement Amendment (CLIA).

Perceived risk of developing colon cancer was considered a cognitive factor mediating the association between receipt of genetic services and resulting variant status with the main outcome, colonoscopy screening. Participants’ perceived risk of developing colon cancer was assessed through response to a single question eliciting what they believe their chance of developing colon cancer was relative to other people their age. Responses were collected on a 5-point scale that ranged from 1 (Much less) to 5 (Much more) with 3 (About the same) being the neutral response.

Provider recommendation for colonoscopy was also considered as a potential mediator between receipt of genetic services and resulting variant status with the main outcome, colonoscopy screening. Provider recommendation for colonoscopy was assessed through a single ‘Yes/No’ question eliciting whether any of their doctors recommended that they undergo a colonoscopy.

Covariates considered in the reported analyses included demographic characteristics, personal cancer history and psychological wellbeing. Demographic variables included age, gender, employment status, health insurance coverage and education level. Personal history of cancer (affected or not affected and type of cancer) was determined by review of medical records for GT family members and self-reported for NGT family members. Psychological wellbeing was assessed through participants’ completion of a short form of the Center for Epidemiology Studies—Depression (CESD) scale. Total CESD scores reflect the summed response across items. Four participants were missing at most a single response on the CESD item set; mean imputation was used to infer these missing responses prior to constructing the total CESD score.

### Statistical analysis

To account for possible clustering of individuals within families, generalized estimating equations (GEE) with an exchangeable covariance structure were used. All multivariate analyses included the aforementioned covariates and used robust standard errors in the computation of significance tests. Three models were fitted. In the first, we test whether NGT participants are less likely to uptake colonoscopy screening than GT+ (Model 1). We then investigate whether inclusion of provider referral to colonoscopy and perceived risk account for observed differences in testing status. In Model 2a, we add provider referral, and in Model 2b, we add perceive cancer risk.
